# Jacobsen Syndrome With White Matter Abnormalities: A Case Report and MRI Follow-Up

**DOI:** 10.7759/cureus.95238

**Published:** 2025-10-23

**Authors:** Hussein W Khudhur, Ghufran Saeed, Fatmah Al Zeyoudi, Amjad M Mohamadiyeh

**Affiliations:** 1 Department of Radiology, Sheikh Khalifa Medical City, Abu Dhabi, ARE; 2 Department of Emergency Medicine, Sheikh Tahnoon Medical City, Al Ain, ARE

**Keywords:** chromosome 11q deletion, genotype-phenotype correlation, jacobsen syndrome, neurodevelopmental delay, white matter abnormalities

## Abstract

Jacobsen syndrome (JBS) is a rare genetic disorder caused by a terminal deletion on chromosome 11q. It is associated with craniofacial dysmorphisms, congenital anomalies, hematological abnormalities, and neurodevelopmental delays. White matter abnormalities (WMAs) are a less commonly reported feature of JBS, with limited cases documenting their progression via MRI follow-ups. This case report highlights a neonate with JBS presenting with a WMA and correlates the imaging findings with clinical improvement.

We report the case of a female neonate born at 37 weeks of gestation with multiple congenital anomalies. She presented with jaundice, respiratory distress, thrombocytopenia, and a systolic murmur. Echocardiography revealed an atrial septal defect and a ventricular septal defect. Dysmorphic features included low-set ears, a depressed nasal bridge, and a prominent philtrum. The patient exhibited left-sided sensorineural hearing loss, hypotonia, and developmental delays. Genetic testing confirmed a terminal deletion at 11q24.1q25, consistent with JBS.

The initial MRI demonstrated unmyelinated white matter in the parieto-occipital lobes without structural abnormalities. Ten months later, a follow-up MRI revealed significant improvement in white matter myelination, which correlated with improved developmental and psychomotor outcomes. WMAs in JBS are believed to result from intra-myelinic edema associated with deletions in genes such as HEPACAM/GlialCAM. Early MRI and serial imaging can document evolving WMAs in JBS and may correlate with clinical improvement, and multidisciplinary follow-up is recommended.

## Introduction

Jacobsen syndrome (JBS) is a rare terminal 11q deletion syndrome characterized by congenital anomalies, thrombocytopenia, and neurodevelopmental delay [1]. The most observed clinical features are abnormalities of the face and skeleton, cognitive impairment, and congenital defects affecting the central nervous system, such as white matter abnormalities (WMAs), heart, urogenital system, gastrointestinal tract, hematological disorders, and even malignancies [2]. Occurrences of ocular, auditory, and immunological abnormalities are also reported in some cases [3]. To date, only slightly more than 200 cases of JBS have been reported. Statistics indicate an incidence of 1 in 100,000 births, with a female-to-male ratio of 2:1 [4,5]. The cause, underlying mechanisms, and progression of WMAs in JBS are not yet fully understood, mainly due to the rarity of reported cases involving WMAs. In this report, we are presenting a case of a neonate with JBS who presented with jaundice, respiratory distress, and thrombocytopenia upon birth and later developed symptoms of congenital heart disease and developmental delays. Our findings contribute to the growing understanding of WMAs in JBS by providing detailed MRI evidence of hypomyelination and its potential progression over time. This report expands current knowledge on the neuroimaging characteristics of the disorder, which may aid in future diagnostic and prognostic assessments.

## Case presentation

A female neonate born at 37 weeks of gestation presented with multiple congenital anomalies, thrombocytopenia, and developmental delays; genetic testing confirmed JBS (11q24.1q25 deletion), and serial MRI documented progressive improvement in white matter myelination correlating with clinical improvement. The neonate was delivered via normal vaginal delivery, with APGAR scores of 8 and 9 at 1 and 5 minutes, respectively. Immediately following birth, the neonate developed respiratory distress, was placed on continuous positive airway pressure (CPAP), and subsequently weaned off to a nasal cannula and then to room air.

Within the first 24 hours of life, the neonate developed significant jaundice, requiring phototherapy. Laboratory investigations revealed elevated direct bilirubin levels and thrombocytopenia (Table 1) that normalized before discharge. Given the respiratory distress and thrombocytopenia, sepsis was initially suspected. A sepsis workup was conducted, and the patient was transferred to the neonatal intensive care unit (NICU) for close monitoring.

**Table 1:** Key neonatal laboratory results

Test	Result	Units	Normal Range
Platelets	111	×10⁹/L	150-400
Total Bilirubin	100.4	μmol/L	<85
Direct Bilirubin	11.4	μmol/L	<5

Upon physical examination, the neonate exhibited a prominent philtrum. Auscultation of the chest revealed a systolic murmur, prompting an echocardiographic evaluation. Echocardiography identified a small ventricular septal defect (VSD) and a moderate atrial septal defect (ASD). The neonate also failed the hearing test for the left ear.

After six days of care in the NICU, which included multiple sessions of phototherapy, the jaundice resolved, platelet levels normalized, and a diagnosis of sepsis was ruled out. The patient was saturating well on room air but was noted to have progressively slower feeding. An abdominal X-ray, conducted to investigate feeding issues, revealed no significant bowel abnormalities but incidentally identified hemivertebrae and anomalous sacral bones.

At three months of age, the patient was brought to the emergency department due to noisy breathing while feeding. Video fluoroscopy revealed aspiration of both thin and thick fluids, indicating unsafe feeding. Chronic nasogastric tube placement was recommended.

During follow-up at five months of age, multiple developmental delays were observed. The patient exhibited poor visual fixation and limited head control, did not startle to loud sounds, and did not reach for objects. Subtle dysmorphic features, including low-set ears and a depressed nasal bridge, were noted in addition to the prominent philtrum previously observed.

Hearing loss was further evaluated by an otolaryngologist. Laryngoscopy identified a laryngeal cleft, and subsequent auditory brainstem response testing revealed severe left-sided sensorineural hearing loss.

**Figure 1:**
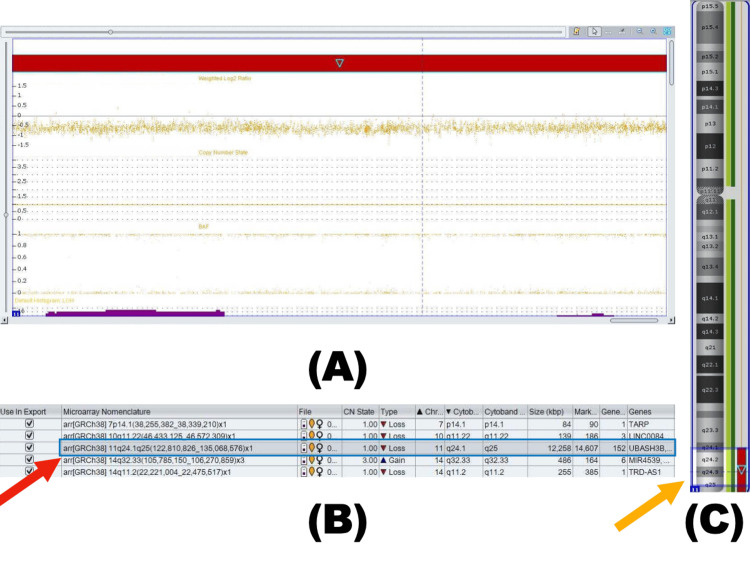
Chromosomal microarray findings in a patient with an 11q terminal deletion (A) Chromosomal microarray (CMA) plot showing weighted log2 ratio, B-allele frequency (BAF), and copy number state (CNS). (B) Copy number variation (CNV) table, identifying a pathogenic heterozygous terminal deletion at 11q24.1-q25 (12,258 kb) (red arrow). (C) Chromosome ideogram, mapping the deleted region on chromosome 11 (yellow arrow).

Given the diversity of symptoms present since birth, a genetic disorder was suspected. Chromosomal microarray was performed at six months of age and identified a terminal heterozygous deletion at 11q24.1-q25 (12,258 kb), consistent with JBS (Figure 1). This diagnosis aligned with the clinical phenotype. The family was counseled regarding the de novo nature of the deletion, recurrence risk, long-term developmental and cardiac implications, and the need for multidisciplinary follow-up; parental testing was not pursued.

**Figure 2:**
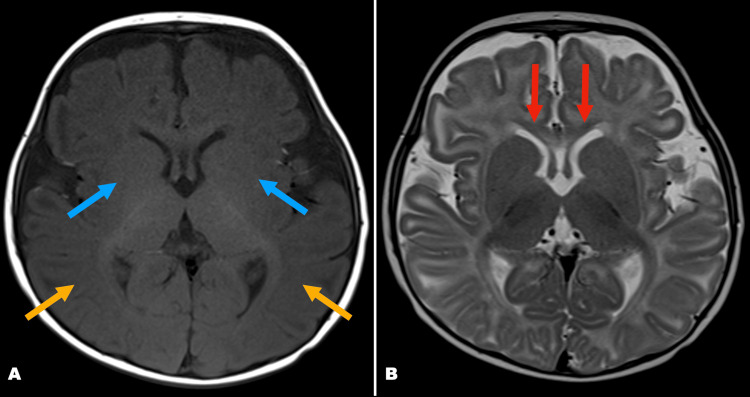
MRI findings of white matter hypomyelination Axial T1 (A) and T2 (B) images at six months demonstrating hypomyelination in parieto-occipital white matter (yellow arrows) and genu of corpus callosum (red arrows), as well as reduced conspicuity of basal ganglia (blue arrows).

As part of the investigations into hypotonia and developmental delay, a magnetic resonance imaging (MRI) scan was performed a few days after the chromosomal microarray analysis. MRI brain was performed on a 1.5T scanner using axial T1-weighted, T2-weighted, FLAIR, and DWI/ADC sequences. Axial T2, as well as axial T1, shows diffuse delayed myelination predominantly in the parieto-occipital white matter and genu of corpus callosum; there was no structural malformation (Figure 2).

**Figure 3:**
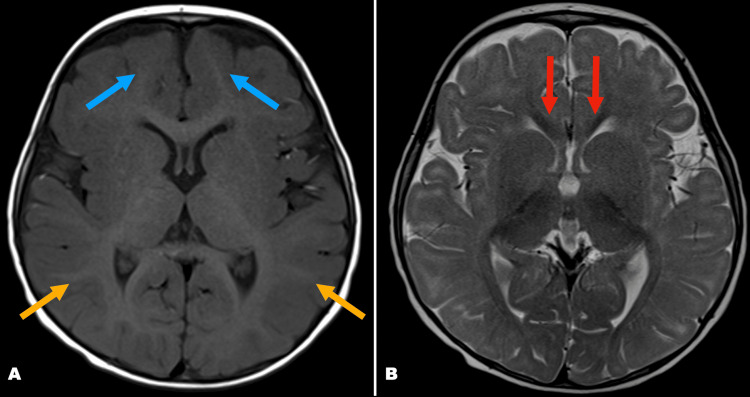
MRI findings indicating improved myelination Axial T1 (A) and T2 (B) at 16 months showing interval improvement in myelination of the corpus callosum (red arrows), parieto-occipital lobes (yellow arrows), and other brain white matter structures (blue arrows).

A second MRI was performed at 16 months of age at the request of the otolaryngologist to assess for nerve aplasia or abnormalities of the internal auditory meatus. This follow-up MRI demonstrated the normal signal intensity and configuration of the inner ear structures, as well as interval improvement in white matter myelination compared to the initial scan (Figure 3).

The patient is currently managed by a multidisciplinary team, including specialists in otolaryngology, pediatric neurology, pediatric gastroenterology, and developmental pediatrics. The parents have been counseled about JBS, the need for regular follow-up visits to address potential complications, and the importance of monitoring developmental milestones.

## Discussion

This case highlights that WMAs can occur in JBS, that serial MRI may document improvement paralleling clinical progress, and that multidisciplinary care is essential for optimal outcomes.

JBS is a genetic disorder associated with developmental delay, psychomotor retardation, and craniofacial dysmorphisms, such as a wide nasal bridge, thin lips, a short nose with anteverted nostrils, retrognathia, ears with a dysmorphic shape and low placement, and camptodactyly. It also involves various malformations affecting the hematological system, causing thrombocytopenia, thrombocytopathy, pancytopenia, and even leukemia. Additionally, JBS is linked to congenital heart defects, gastrointestinal, renal, neurological, and skeletal deformities affecting the skull, face, vertebrae, and limbs [2]. Thrombocytopenia is a significant and common complication in JBS, with a high incidence of 88.5%. Recent studies show that approximately 20% of infants diagnosed with JBS do not survive beyond the first two years of life. In most cases, mortality is primarily due to congenital heart disease, while in some instances, it results from hemorrhage caused by thrombocytopenia [6]. The severity of bleeding will never be detected with the number of platelets, although our patient with a platelet count of 111 × 10⁹/L still can bleed aggressively due to platelet dysfunction and coagulopathy [1].

A study done on rats identified a critical 1.2 Mb region on distal chromosome 11q including RICS, ETS-1, and other genes linked to congenital heart defects [7]. However, another research study suggests that congenital heart diseases are caused by disruption of the area 7 Mb on distal chromosome 11q responsible for congenital heart diseases [3]. In our case, echocardiography revealed both ASD and VSD, consistent with the role of chromosome 11q in cardiac abnormalities.

WMAs in JS have been reported in only a few cases, with limited instances in early infancy assessed by MRI [8,9]. The WMA observed in JS is believed to result from white matter water retention and intra-myelinic edema associated with HEPACAM/GlialCAM deletions, similar to findings in MLC2A and MLC2B patients. Various mutations have been identified, and multiple genes within the deleted region may contribute to the development of WMA [10-12]. A limitation of this study is the rarity of reported cases and limited longitudinal data on WMA in JBS, which restricts the generalizability and strength of prognostic conclusions.

In MLC2A and MLC2B, the causative genes are linked to chronic white matter edema, macrocephaly, and frontotemporal or frontoparietal subcortical cysts. MLC2A is classified as an autosomal recessive disorder, presenting with a more severe phenotype that shows no improvement in psychomotor development or WMA. In contrast, MLC2B is autosomal dominant and typically presents a milder phenotype, with gradual improvement observed in many patients between the ages of 1 and 4 years [10,11].

In our case, the patient’s first MRI, performed at six months of age, revealed unmyelinated white matter in the parieto-occipital lobes without any structural abnormalities. A follow-up MRI at 16 months of age showed interval improvement in white matter myelination compared to the initial scan, which correlated with noticeable progress in developmental delay and psychomotor retardation. In comparison with another case report, which tracked a patient’s development and imaging findings in detail over the study period, demonstrating that while psychomotor development initially showed retardation, it continuously improved alongside the imaging findings. Both cases support the conclusion that improvements in imaging findings are indicative of symptomatic improvement [13].

The observed improvement in WMA suggests that serial MRI may guide tailored rehabilitation strategies in JBS. Given the de novo nature and low recurrence risk, genetic counseling should prepare families for potential developmental trajectories while emphasizing the value of longitudinal neuroimaging in monitoring progress.

## Conclusions

This case highlights the potential link between early WMAs and developmental outcomes in JBS. While our findings suggest that progressive myelination may correlate with clinical improvement, the lack of long-term follow-up limits definitive conclusions. Nevertheless, documenting MRI changes provides valuable insight into the neurodevelopmental changes of affected patients. 

Although JBS presents with diverse clinical features, the genotype-phenotype correlation remains unclear, highlighting the importance of cytogenetic testing for confirmation. Few reports discuss WMAs in JBS with detailed MRI follow-ups, emphasizing the rarity of such findings. Regular multidisciplinary follow-up plays a crucial role in improving motor and intellectual development in JBS patients. Future studies with serial imaging and extended clinical monitoring are essential to clarify prognostic indicators and guide multidisciplinary management in rare genetic syndromes like JBS.
